# N6-methyladenosine epitranscriptomic remodeling drives infantile aortic coarctation via FTO-mediated repression of PTEN and GRK5

**DOI:** 10.3389/fcvm.2026.1882212

**Published:** 2026-08-12

**Authors:** Wenlei Li, Weidan Chen, Minghui Zou, Xun Ma, Xinxin Chen, Huikang Tao, Li Ma

**Affiliations:** 1 Heart Center, Department of Pediatric Surgery, Guangdong Provincial Key Laboratory of Research in Structural Birth Defect Disease, Guangzhou Women and Children’s Medical Center, Guangzhou Medical University, Guangzhou, Guangdong, China; 2 Department of Cardiovascular Surgery, Zhongnan Hospital of Wuhan University, Wuhan, China

**Keywords:** aortic coarctation, aortic fibrosis, epigenetics, FTO, GRK5, infants, m6A RNA methylation, PTEN

## Abstract

**Introduction:**

Aortic coarctation (CoA) is a prevalent congenital heart defect defined by focal aortic narrowing and progressive fibrosis, associated with substantial long-term cardiovascular morbidity. Although surgical repair improves early survival, the molecular and epigenetic mechanisms governing CoA pathogenesis remain poorly understood. N6-methyladenosine (m6A), the most abundant internal mRNA modification, regulates post-transcriptional gene expression during cardiovascular development and disease. In this study, we investigated whether dysregulated m6A epitranscriptome contributes to infantile CoA.

**Methods:**

Global m6A abundance was measured in stenotic aortic tissues and paired normal aortic segments from infants with CoA using dot blot and colorimetric assays. Transcriptome-wide m6A landscapes were profiled by methylated RNA immunoprecipitation sequencing (MeRIP-seq), and mRNA expression patterns were determined by RNA sequencing (RNA-seq). Quantitative real-time PCR (qRT-PCR) was used to validate key genes and m6A regulatory factors. To establish causal regulatory relationships, we further performed FTO loss-of-function knockdown experiments in human aortic smooth muscle cells (HASMCs).

**Results:**

We found that global m6A levels are significantly elevated in CoA tissues, with widespread hypermethylation events enriched in focal adhesion and extracellular matrix remodeling pathways. Integrated multi-omics analysis identified FTO (alpha-ketoglutarate dependent dioxygenase) downregulation as the primary driver of m6A hypermethylation in CoA. Mechanistically, reduced FTO expression increases m6A methylation of phosphatase and tensin homolog (PTEN) and G protein-coupled receptor kinase 5 (GRK5), resulting in their transcriptional repression in stenotic aortic tissue. In HASMCs, silencing FTO directly elevated m6A modification and suppressed PTEN and GRK5 expression. Functionally, diminished PTEN expression promotes aortic smooth muscle fibrosis and luminal narrowing.

**Conclusion:**

Our findings uncover a previously unrecognized FTO/m6A/PTEN epitranscriptomic axis that governs infantile CoA pathogenesis, providing novel epigenetic targets for therapeutic intervention.

## Graphical Abstract

**Figure:**
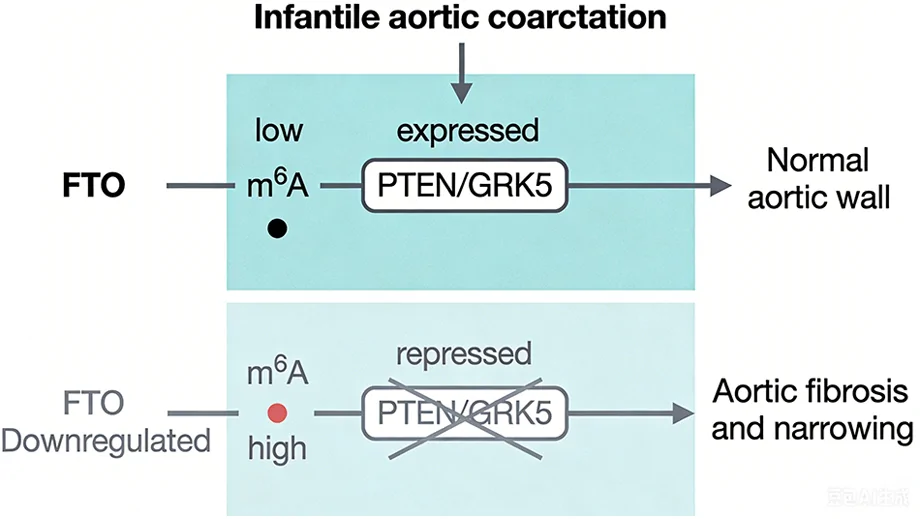
Schematic of the FTO/m⁶A/PTEN-GRK5 axis in infantile aortic coarctation. FTO downregulation increases m⁶A methylation of PTEN and GRK5, repressing their expression and promoting aortic fibrosis and luminal narrowing.

## Introduction

Aortic coarctation (CoA) is a congenital cardiovascular malformation characterized by localized narrowing of the juxtaductal aorta, accounting for 6%–8% of all congenital heart disease cases (1). Pathologically, CoA arises from aberrant migration of ductus arteriosus-derived smooth muscle cells (SMCs) into the aortic wall, followed by excessive extracellular matrix (ECM) deposition and progressive luminal fibrosis (2, 3). While surgical correction reduces early mortality, patients remain at high risk of persistent hypertension, heart failure, aortic aneurysm, and cerebrovascular complications, which significantly impair long-term clinical outcomes. Elucidating the molecular drivers of CoA is therefore essential to identify effective therapeutic strategies. To date, research efforts have focused primarily on surgical techniques and clinical management, with limited progress in defining the epigenetic mechanisms underlying CoA development.

N⁶-methyladenosine (m⁶A) is the most abundant internal modification of eukaryotic mRNA and long non-coding RNA (lncRNA), regulating RNA stability, splicing, translation, and subcellular trafficking (4). The m⁶A landscape is dynamically modulated by methyltransferases (writers: METTL3/METTL14), demethylases (erasers: FTO/ALKBH5), and m⁶A-binding proteins (readers: YTHDF1/2) (5). Dysregulated m⁶A modification has been implicated in multiple cardiovascular disorders, including atherosclerosis, myocardial infarction, and congenital heart defects (6). Notably, m⁶A-dependent epigenetic regulation controls embryonic stem cell differentiation and cardiovascular lineage specification, processes critical to congenital heart disease pathogenesis (7, 8). For example, METTL14 depletion impairs stem cell self-renewal, while ZC3H13 loss accelerates differentiation via reduced m⁶A levels (7, 8). These observations strongly suggest that m⁶A dysregulation may contribute to developmental cardiovascular disorders, including CoA.

Despite its biological relevance, the role of m⁶A epitranscriptomics in infantile CoA remains unexplored. In the present study, we performed an integrated transcriptomic and epitranscriptomic analysis of stenotic aortic tissues and paired normal aortic segments from infants with CoA. We demonstrate that FTO downregulation-mediated m⁶A hypermethylation represses PTEN and GRK5 expression, driving aortic fibrosis and CoA pathogenesis. This work provides the first comprehensive m⁶A methylome landscape of infantile CoA and identifies a novel epigenetic regulator*y* axis with potential therapeutic implications.

## Methods

### Patient cohort and tissue specimens

Twelve neonates with aortic coarctation (CoA) who received surgical treatment at Guangzhou Women and Children’s Medical Center were prospectively recruited in this study.

### Inclusion criteria

Neonates aged ≤120 days, with juxtaductal aortic coarctation confirmed by preoperative echocardiography or computed tomography angiography, requiring surgical resection of stenotic aortic tissues; Complete clinical baseline data including age, body weight, gender and concomitant cardiac malformations; Legal guardians voluntarily signed written informed consent for specimen collection and experimental research; Intact stenotic aortic tissue and matched normal aortic segments distal to the stenosis could be harvested during surgery.

### Exclusion criteria

Patients with acquired aortic stenosis induced by trauma, autoimmune vasculitis or postoperative adhesions; Combined severe systemic genetic disorders, autoimmune diseases or multi-organ dysfunction; Intraoperative damaged, incomplete or degraded tissues unsuitable for subsequent molecular detection and sequencing; Guardians declined to provide tissue samples for research purposes.

The research protocol was reviewed and approved by the Institutional Review Board of Guangzhou Women and Children's Medical Center (approval number: [2021]244A01). Preoperative imaging examinations were implemented for all subjects to confirm CoA diagnosis, and clinical information was retrospectively extracted from electronic medical records, with demographic data summarized in Table 1. During operation, juxtaductal stenotic aortic tissues were collected as CoA groups, and histologically normal aortic tissues distal to the narrow segment were taken as paired control samples without interfering with surgical procedures. All tissues were immediately snap-frozen in liquid nitrogen and preserved at −80 °C for subsequent experiments. High-throughput RNA-seq and MeRIP-seq analyses were conducted on six pairs of specimens (patient No.7–12), while dot blot, colorimetric m6A quantification and qRT-PCR validation experiments used tissues from patients 1–6.

**Table 1:** Clinical characteristics of enrolled patients.

Patients	Sex	Age (day)	Body weight (kg)	Associated lesions
1	M	11	2.4	PDA, PFO
2	M	11	3.4	VSD, ASD, PDA
3	M	35	3.6	VSD, ASD, PDA
4	M	7	2.54	PDA, PFO
5	M	18	3.5	VSD, ASD
6	M	16	4.2	VSD, PFO
7	F	12	3.6	VSD, PDA, ASD
8	M	52	4.5	VSD
9	M	117	4.7	VSD
10	M	28	3.9	NONE
11	M	13	3.2	VSD, PDA, PFO
12	F	91	5	VSD, MR, PDA
Total	F/M (2/1)	34.3 ± 33.9	3.7 ± 0.8	

VSD, ventricular septal defect; PDA, patent ductus arteriosus; PFO, patent foramen ovale; MR, mitral regurgitation; ASD, atrial septal defect; Note that samples of high-throughput sequencing were obtained from patients 7–12.

### Global m⁶A quantification

Total RNA was extracted from frozen aortic tissues using TRIzol reagent (Thermo Scientific) following standard protocols. For dot blot analysis, 1 μg purified RNA was spotted onto nitrocellulose membranes, cross-linked at 65 °C for 30 min and blocked with 5% non-fat TBST buffer for 1 h at room temperature. Membranes were incubated with anti-m⁶A primary antibody (1:5000) overnight, followed by three rounds of TBST washing, and incubated with HRP-conjugated secondary antibody (1:20000) for 1 h. Chemiluminescence signals were captured for visualization. For colorimetric m⁶A detection, 200 ng total RNA was measured with EpiQuik m⁶A RNA Methylation Quantification Kit (Abcam) according to manufacturer's instructions, and absorbance at 450 nm was recorded to calculate relative m⁶A levels via standard curve.

### RNA-seq and MeRIP-seq

Poly(A) mRNA was isolated via Arraystar Seq-Star™ Kit. Purified mRNA was fragmented to ∼100 nt; one portion was enriched by anti-m⁶A antibody for MeRIP, and the remaining RNA served as input control. Library construction was performed using KAPA Stranded mRNA-seq Kit, followed by sequencing on Illumina HiSeq 4000 platform. Raw sequencing reads were quality-controlled with FastQC and trimmed by Trimmomatic, then aligned to human hg38 reference genome using HISAT2. Differentially methylated m⁶A peaks were screened with ExomePeak (*p* < 0.05). Subsequent peak annotation, transcript distribution analysis, motif enrichment and multi-omics combined analysis were performed.

### Quantitative real-time PCR (qRT-PCR)

#### Tissue RNA qRT-PCR

Total RNA was extracted from frozen aortic tissues by TRIzol reagent. RNA purity and concentration were detected with Nanodrop 2000, and RNA integrity was verified by agarose gel electrophoresis. First-strand cDNA was synthesized from 1 μg total RNA using PrimeScript RT Kit (Takara). qRT-PCR amplification was carried out on Accurate 96 real-time PCR system with TB Green Premix Ex Taq II, and GAPDH was used as internal reference gene. Relative mRNA expression was calculated by the 2^−ΔΔCt method.

#### Cellular RNA qRT-PCR

Total RNA was extracted from sh-NC and sh-FTO stable HASMC lines. Reverse transcription and amplification procedures were consistent with tissue samples. Detected transcripts included m⁶A regulators (FTO, METTL3, METTL14, ALKBH5, YTHDF1, YTHDF2), target genes (PTEN, GRK5) and fibrotic markers (COL1A1, COL3A1, ACTA2). All primer sequences for tissue and cell detection were listed in Supplementary Table S1. Each sample was set with three technical replicates, and three independent biological repeats were conducted for cell experiments. Primer sequences are listed in Supplementary Table S1.

#### Cell culture of human aortic smooth muscle cells (HASMCs)

Primary human aortic smooth muscle cells (HASMCs, Cat. JY-J2024, Jinyuan Biotechnology) were used for *in vitro* functional verification. Cells were cultured in smooth muscle cell complete medium supplemented with 5% fetal bovine serum, smooth muscle cell growth supplement and penicillin/streptomycin (100 U/mL penicillin, 100 μg/mL streptomycin). The culture environment was maintained at 37 ^o^C with 5% CO_2_ and saturated humidity. Cells at passages 3 were utilized for all transfection and detection experiments to avoid phenotypic drift. Culture medium was refreshed every 48 h.

#### Lentiviral shRNA transfection for stable FTO knockdown

Short hairpin RNA (shRNA) targeting human FTO (sh-FTO) and non-target negative control shRNA (sh-NC) were constructed into the pLKO.1 lentiviral vector by GenePharma Co., Ltd. Lentiviral packaging plasmids psPAX2 and pMD2.G were co-transfected into 293 T cells to produce infectious lentivirus supernatant. Virus-containing medium was collected at 48 h and 72 h post-transfection, filtered through a 0.45 μm filter, and concentrated by ultracentrifugation.

For HASMC transduction, cells were seeded in 6-well plates until reaching 60%–70% confluency. Polybrene (8 μg/mL) was added to improve lentiviral infection efficiency, followed by incubation with sh-FTO or sh-NC lentivirus for 12 h. After viral medium replacement with fresh complete medium, puromycin (2 μg/mL) was added for continuous screening for 7 days to establish stable FTO knockdown cell lines. Stably transfected cell pools were maintained in medium containing 1 μg/mL puromycin for subsequent functional assays.

#### Western blot

Total protein was extracted from stable sh-NC and sh-FTO transfected HASMCs using RIPA lysis buffer containing 1 mM phenylmethylsulfonyl fluoride (PMSF) and protease inhibitor cocktail (Beyotime, China). Protein concentration was quantified via BCA protein quantification kit. Equal amounts of total protein were separated by 10% SDS-PAGE gel and transferred onto PVDF membranes. After blocking with 5% skim milk at room temperature for 1.0 h, membranes were incubated with primary antibodies overnight at 4 ^o^C: anti-FTO (27226-1-AP, proteintech,1:1000), anti-PTEN (60300-3-Ig, proteintech,1:1000), anti-GRK5 (17032-1-AP, proteintech,1:1000), and anti-GAPDH (GB15002, Servicebio,1:10000) as internal reference. After three TBST washes, membranes were incubated with HRP-conjugated secondary antibody (GB150157, Servicerbio, 1:8000) at room temperature for 1 h. Protein bands were visualized using enhanced chemiluminescence (ECL) reagent, and band gray values were quantified by ImageJ software. Relative protein expression was normalized to GAPDH levels, with three independent biological replicates performed for all groups.

### Statistical analysis

Data are presented as mean ± standard deviation. Group comparisons were performed using Student's t-test,no multiple groups were compared in this study. Statistical significance was defined as *p < 0.05, **p < 0.01, ***p < 0.001. Analyses were conducted with GraphPad Prism 9.0.

## Results

### Global m⁶A hypermethylation is a hallmark of infantile CoA

**Figure 1:**
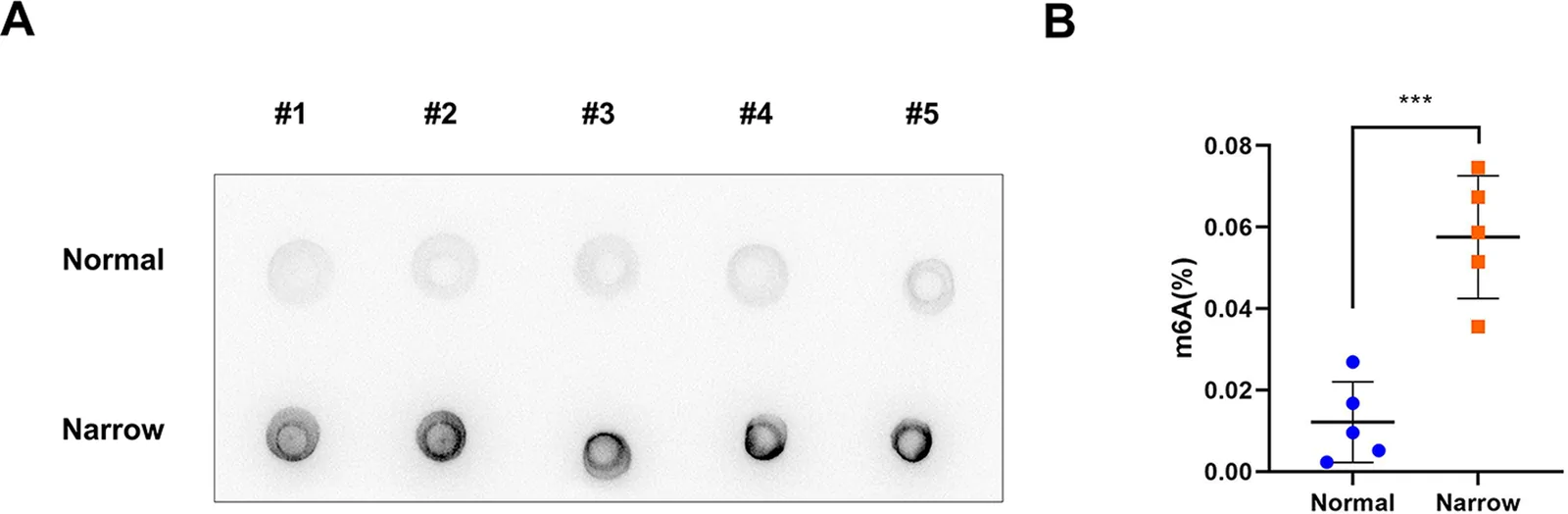
Global m⁶A hypermethylation in infantile CoA **(A)** Dot blot analysis of m⁶A levels in control and CoA tissues. **(B)** Colorimetric quantification of global m⁶A abundance. Data: mean ± SD (*N* = 5/group). ***p < 0.001 vs. control.

To define the m⁶A landscape in CoA, we quantified global m⁶A levels in 5 paired CoA and control aortic tissues. Dot blot analysis revealed markedly increased m⁶A signal intensity in CoA tissues compared with controls (Figure 1A). Colorimetric quantification confirmed a significant elevation of total m⁶A levels in CoA tissues (*N* = 5/group; Figure 1B). These results establish m⁶A hypermethylation as a key epigenetic feature of infantile CoA.

### Widespread transcriptomic dysregulation in CoA tissues

**Figure 2:**
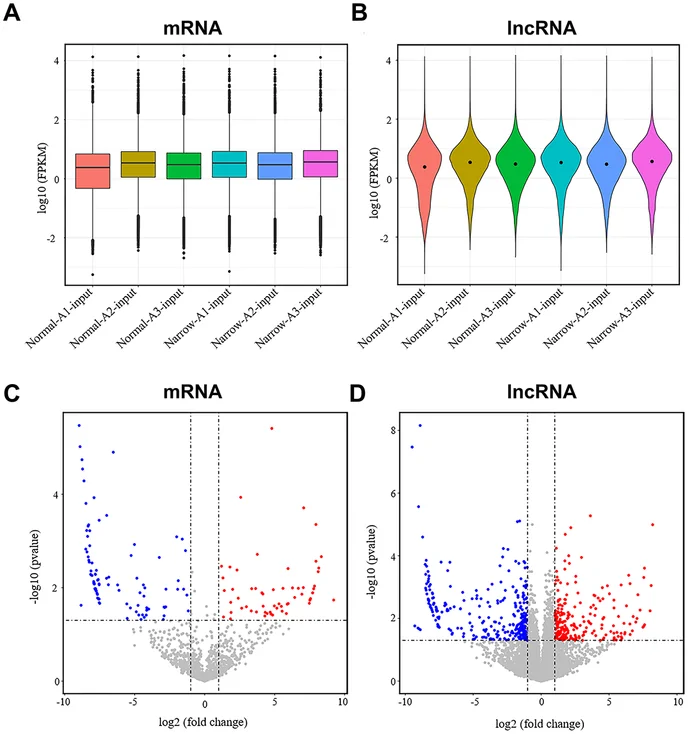
Transcriptomic dysregulation in CoA. **(A)** Box plot and **(B)** violin plot of gene expression distribution (FPKM normalization). Volcano plots of differentially expressed **(C)** lncRNAs and **(D)** mRNAs. Statistical analysis: unpaired Student's t-test. Red: upregulated; Blue: downregulated (fold change > 2, *N* = 3/group).

RNA-seq was performed to identify differentially expressed transcripts between CoA and control aortic tissues. A total of 15,043 transcripts, including 13,685 mRNAs and 1,358 lncRNAs, were detected. FPKM normalization confirmed distinct gene expression profiles between groups (Figures 2A,B). Volcano plots revealed 27 differentially expressed lncRNAs and 40 mRNAs (Figures 2C,D, Supplementary Table S2, S3).

### GO/KEGG enrichment implicates fibrosis and adhesion pathways

**Figure 3:**
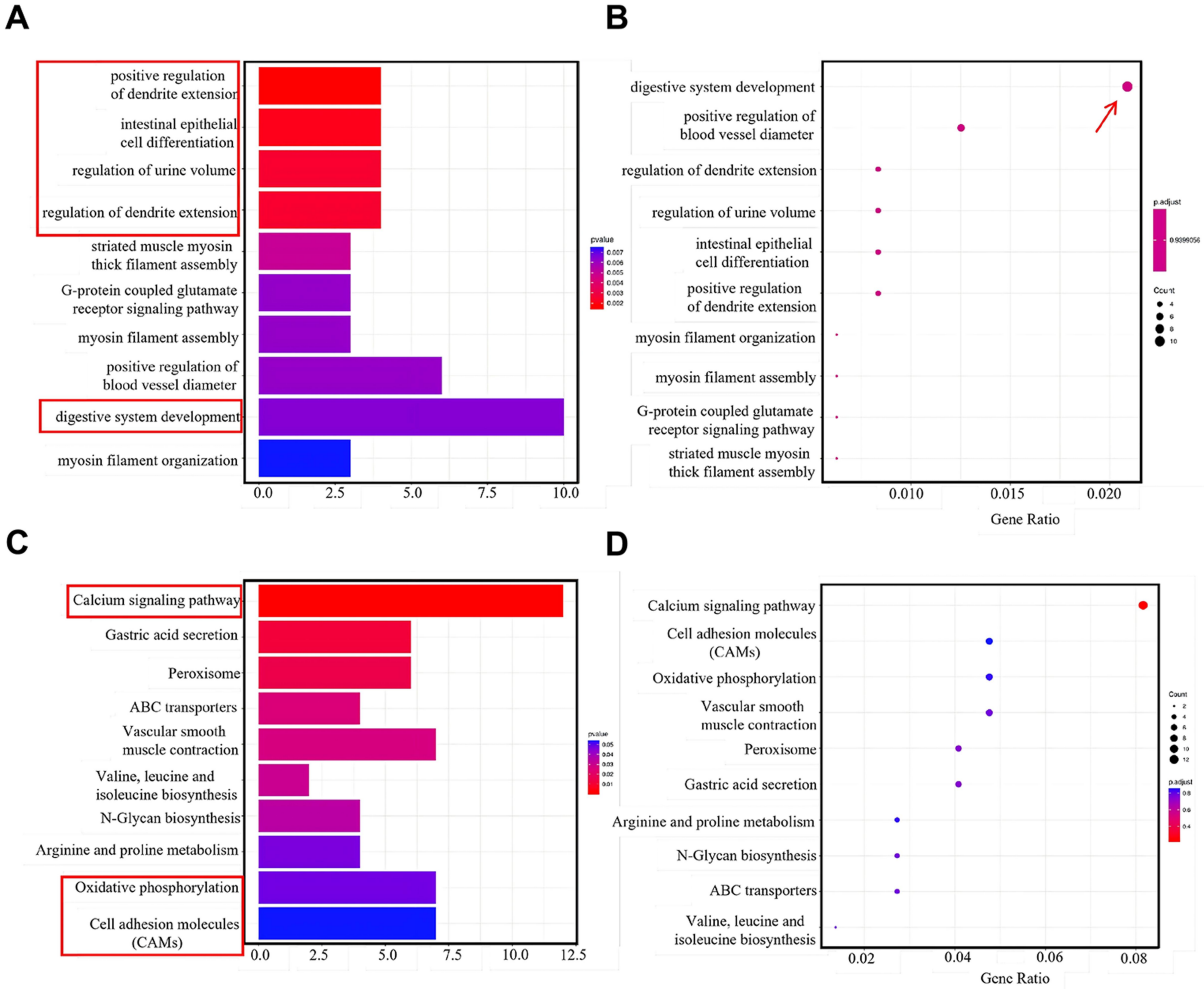
GO and KEGG enrichment of differentially expressed mRNAs. **(A,B)** GO biological process enrichment (bar and dot plots). **(C,D)** KEGG pathway enrichment (bar and dot plots).Statistical test threshold: *p* < 0.05 for enriched pathways.

GO analysis of differentially expressed mRNAs revealed enrichment in developmental processes, including digestive system development and cell adhesion (Figures 3A,B). Kyoto Encyclopedia of Genes and Genomes (KEGG) pathway analysis identified significant enrichment in calcium signaling, oxidative phosphorylation, and cell adhesion molecule pathways (Figures 3C,D).

### Transcriptome-wide m⁶A methylome remodeling in CoA

**Figure 4:**
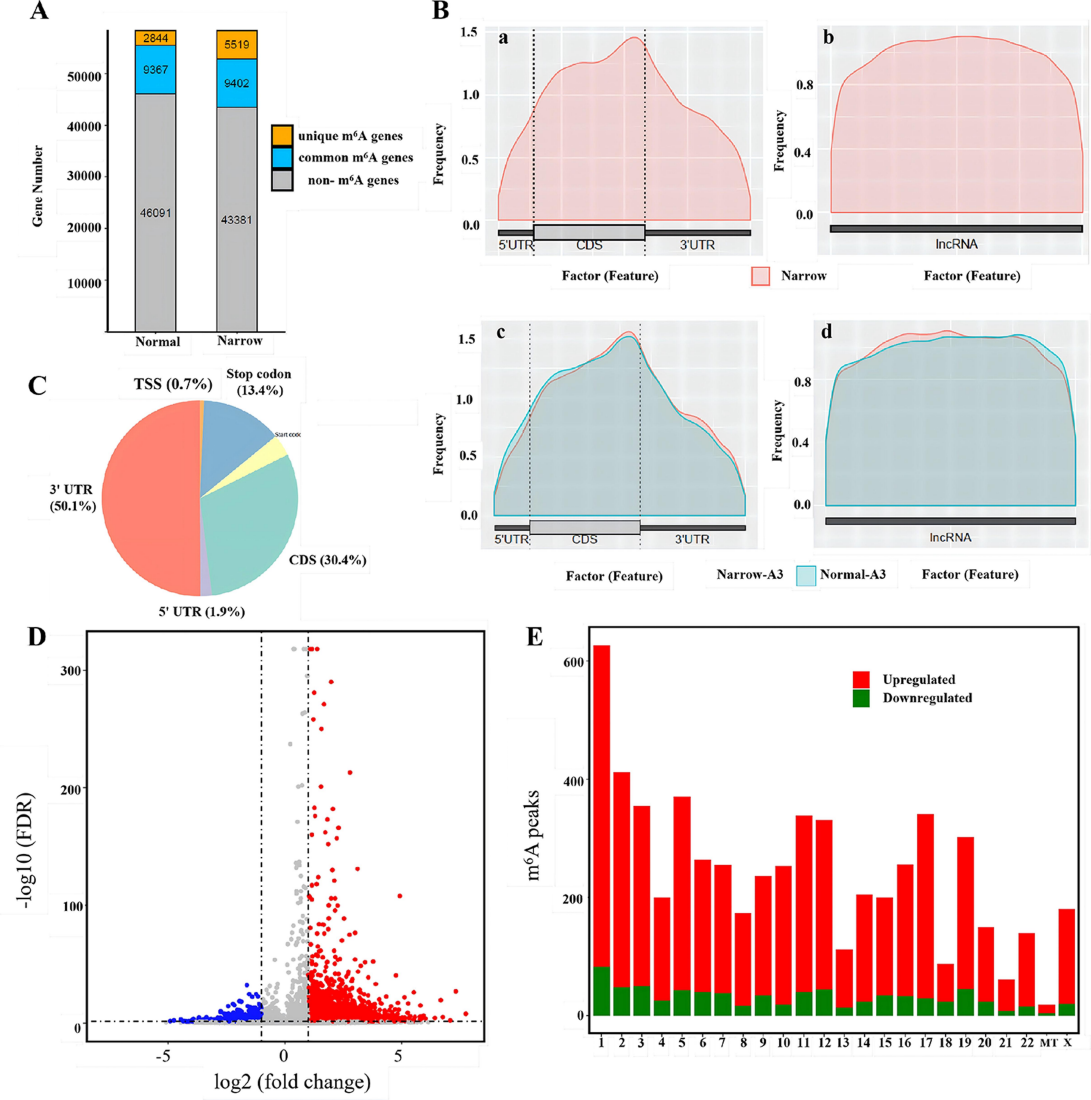
Transcriptome-wide m⁶A methylome remodeling in CoA. **(A)** Venn diagram of m⁶A-modified genes. **(B)** Metagene plots of m⁶A distribution. **(C)** Pie chart of m⁶A peak genomic distribution. **(D)** Volcano plot of differentially methylated m⁶A peaks. **(E)** Chromosomal distribution of differential m⁶A peaks. Differential m⁶A peaks were identified by ExomePeak with *p* < 0.05 threshold.

MeRIP-seq identified 5,519 unique m⁶A-modified genes in CoA tissues versus 2,844 in controls, indicating CoA-specific m⁶A events (Figure 4A). Metagene plots confirmed canonical m⁶A distribution: peaks near the CDS/3’UTR boundary in mRNAs and uniform across lncRNAs (Figure 4B). Global peaks were enriched in 3’UTR (50.1%), CDS (30.4%), and stop codon (13.4%) regions (Figure 4C).

Volcano plots identified widespread m⁶A hypermethylation in CoA tissues (fold change > 2; Figure 4D). Dysregulated m⁶A peaks were distributed across all human chromosomes, with the highest abundance on chromosomes 1, 2, and 5, and consistently more upregulated than downregulated peaks (Figure 4E). Top differential m⁶A peaks identified from MeRIP-seq data are listed in Supplementary Table S4 (lncRNA) and Supplementary Table S5 (mRNA).

### M⁶A hypermethylation enriches in focal adhesion signaling

**Figure 5:**
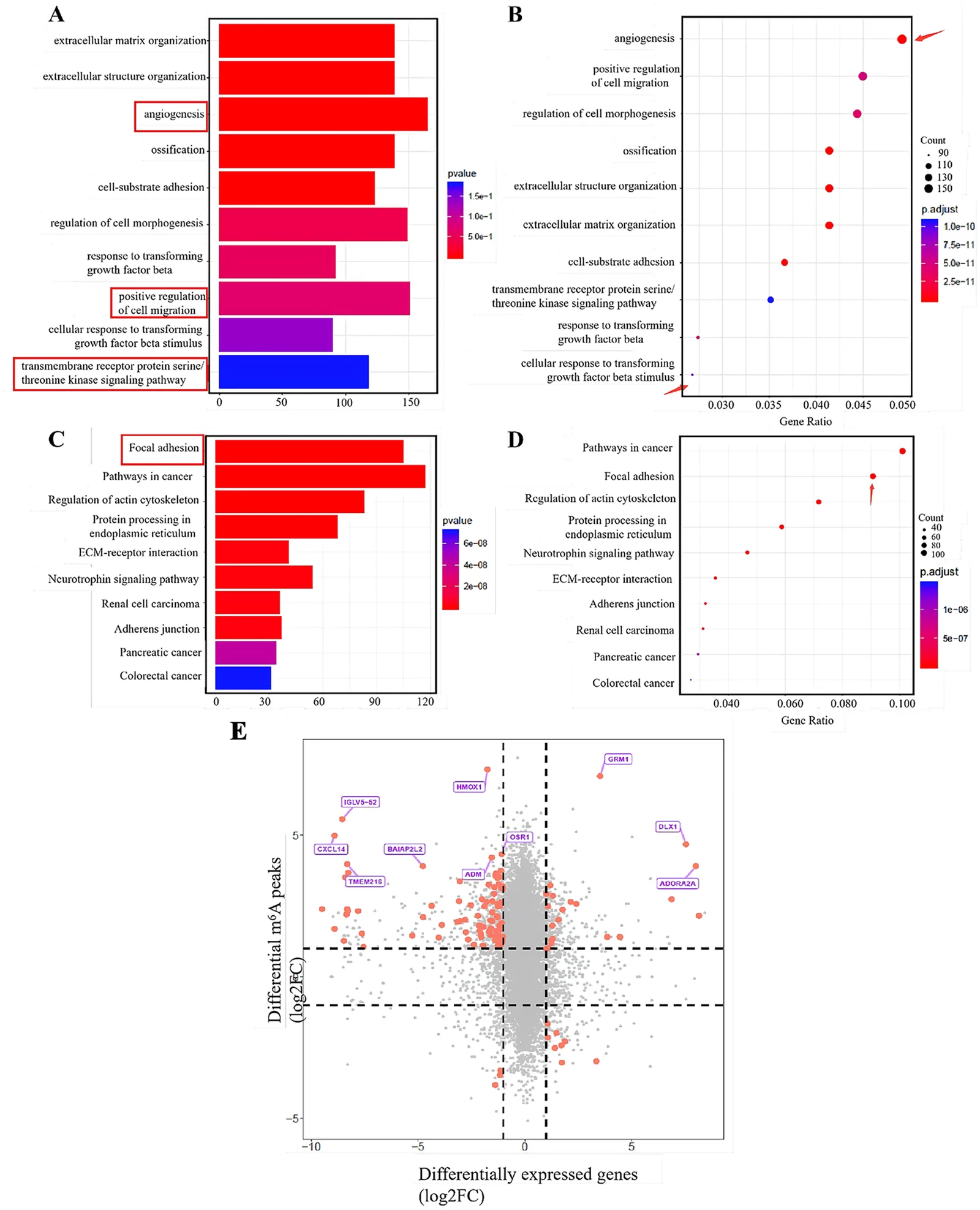
Pathway enrichment and integrated multi-omics analysis. **(A,B)** KEGG enrichment of m⁶A-modified transcripts. **(C,D)** KEGG enrichment of differentially methylated m⁶A transcripts. **(E)** Four-quadrant plot of integrated MeRIP-seq and RNA-Seq data, highlighting top 10 candidate transcripts.

KEGG analysis of m⁶A-modified transcripts revealed enrichment in angiogenesis, cell migration, and focal adhesion signaling (Figures 5A–D), a pathway critical for SMC adhesion, migration, and ECM remodeling.Four-quadrant plot analysis identified 10 candidate transcripts with m⁶A hypermethylation and differential expression. GRM1/DLX1/ADORA2A were upregulated; HMOX1/IGLV5−52/CXCL14/ BAIAP2L2/OSR1/TMEM216/ADM were downregulated (Figure 5E). HMOX1, linked to aortic aneurysm and hypertension, may contribute to CoA complications.

### FTO downregulation drives m⁶A hypermethylation and represses PTEN/GRK5

**Figure 6:**
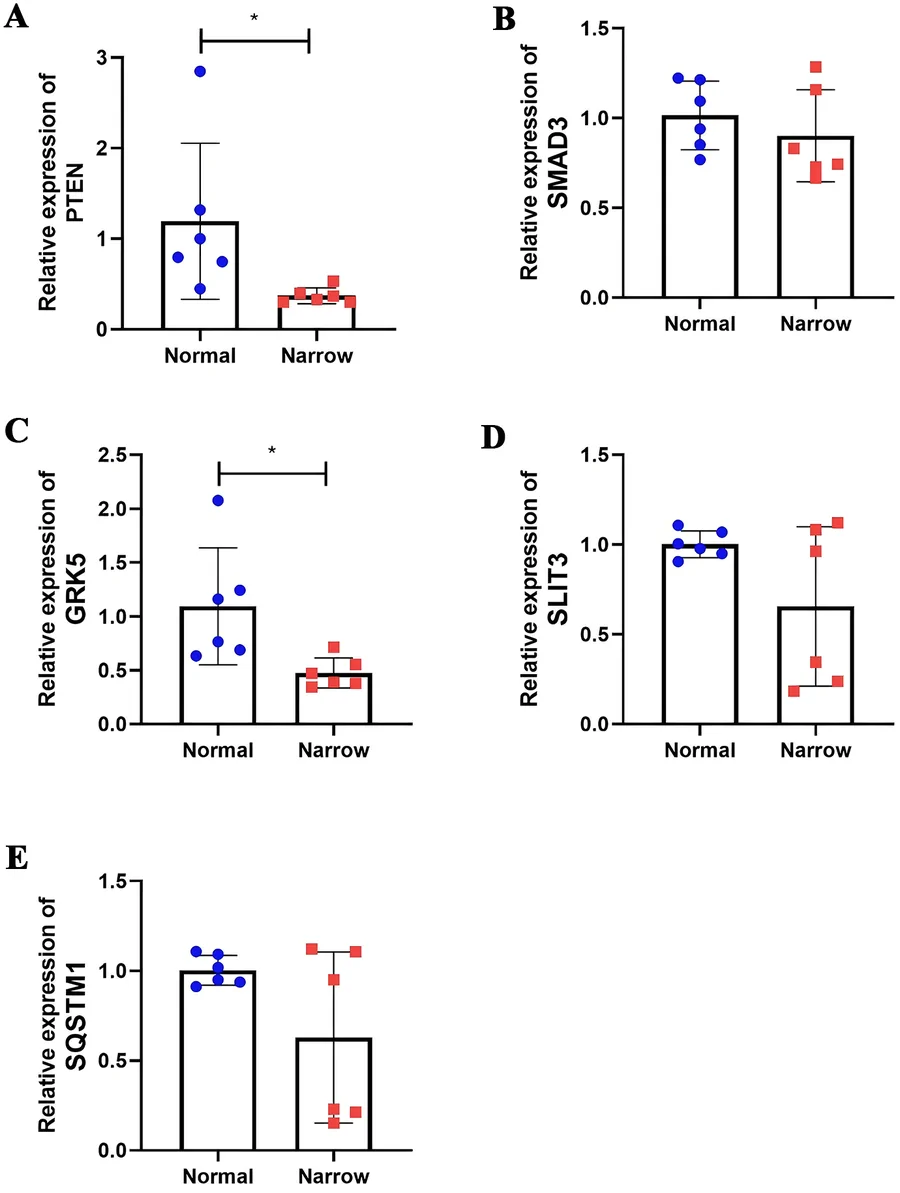
Validation of CoA-associated m⁶A-modified genes. qRT-PCR analysis of **(A)** PTEN, **(B)** SMAD3, **(C)** GRK5, **(D)** SLIT3, and **(E)** SQSTM1 expression. Data: mean ± SD (*N* = 3/group). *p < 0.05 vs. control.

**Figure 7:**
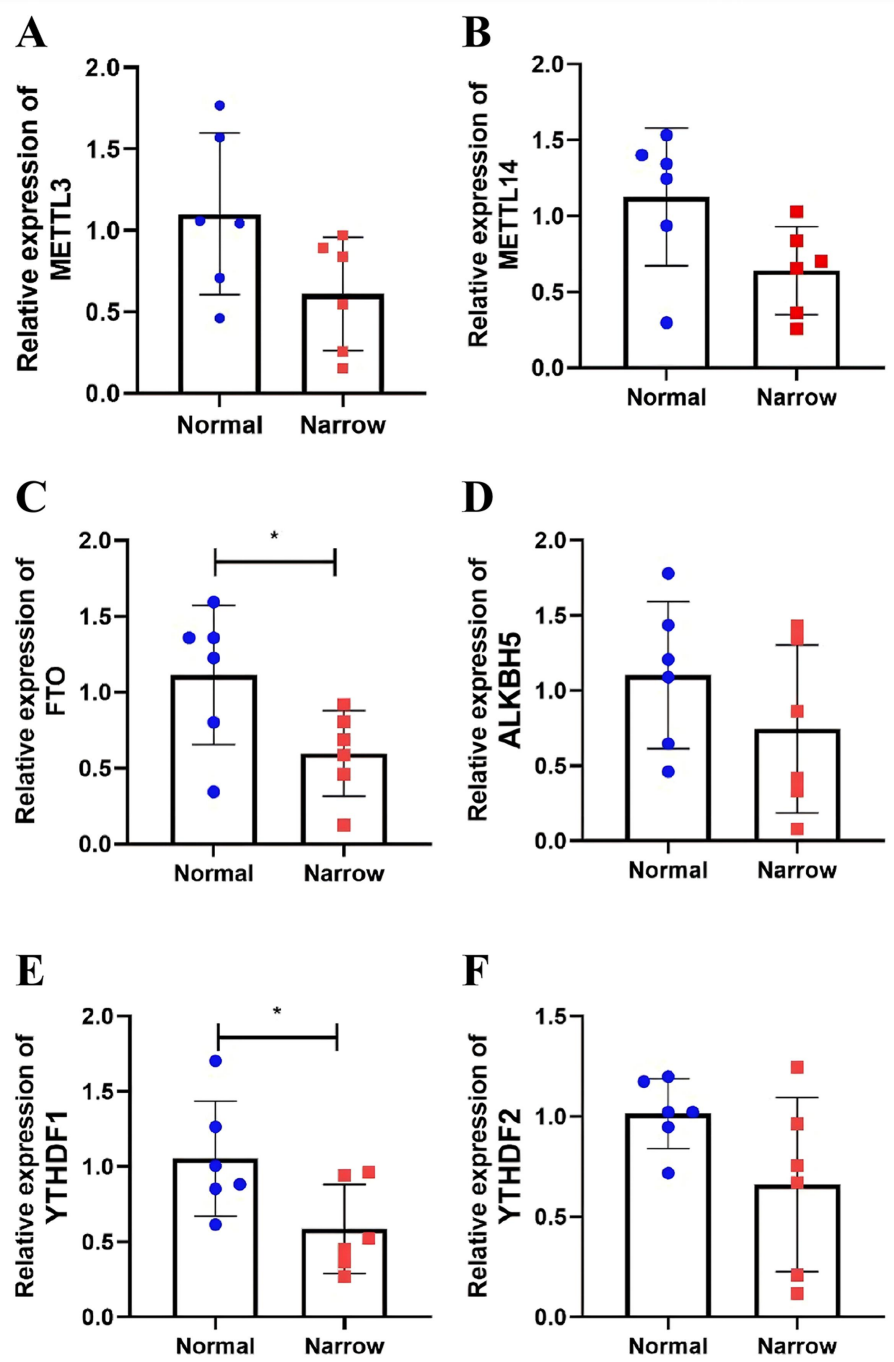
Expression of m⁶A regulatory machinery. qRT-PCR analysis of **(A)** METTL3, **(B)** METTL14, **(C)** FTO, **(D)** ALKBH5, **(E)** YTHDF1, and **(F)** YTHDF2 expression. Data: mean ± SD (*N* = 3/group). *p < 0.05 vs. control.

qRT-PCR validated significant downregulation of PTEN and GRK5 in CoA tissues (Figures 6A,C), with no changes in SMAD3/SLIT3/SQSTM1 (Figures 6B–E). Among m⁶A regulators, FTO and YTHDF1 were significantly downregulated in CoA (Figures 7C,E), while METTL3/METTL14/ ALKBH5/YTHDF2 remained unchanged (Figures 7A–F). These data identify FTO deficiency as the primary driver of m⁶A hypermethylation, leading to PTEN/GRK5 repression in CoA.

**Figure 8:**
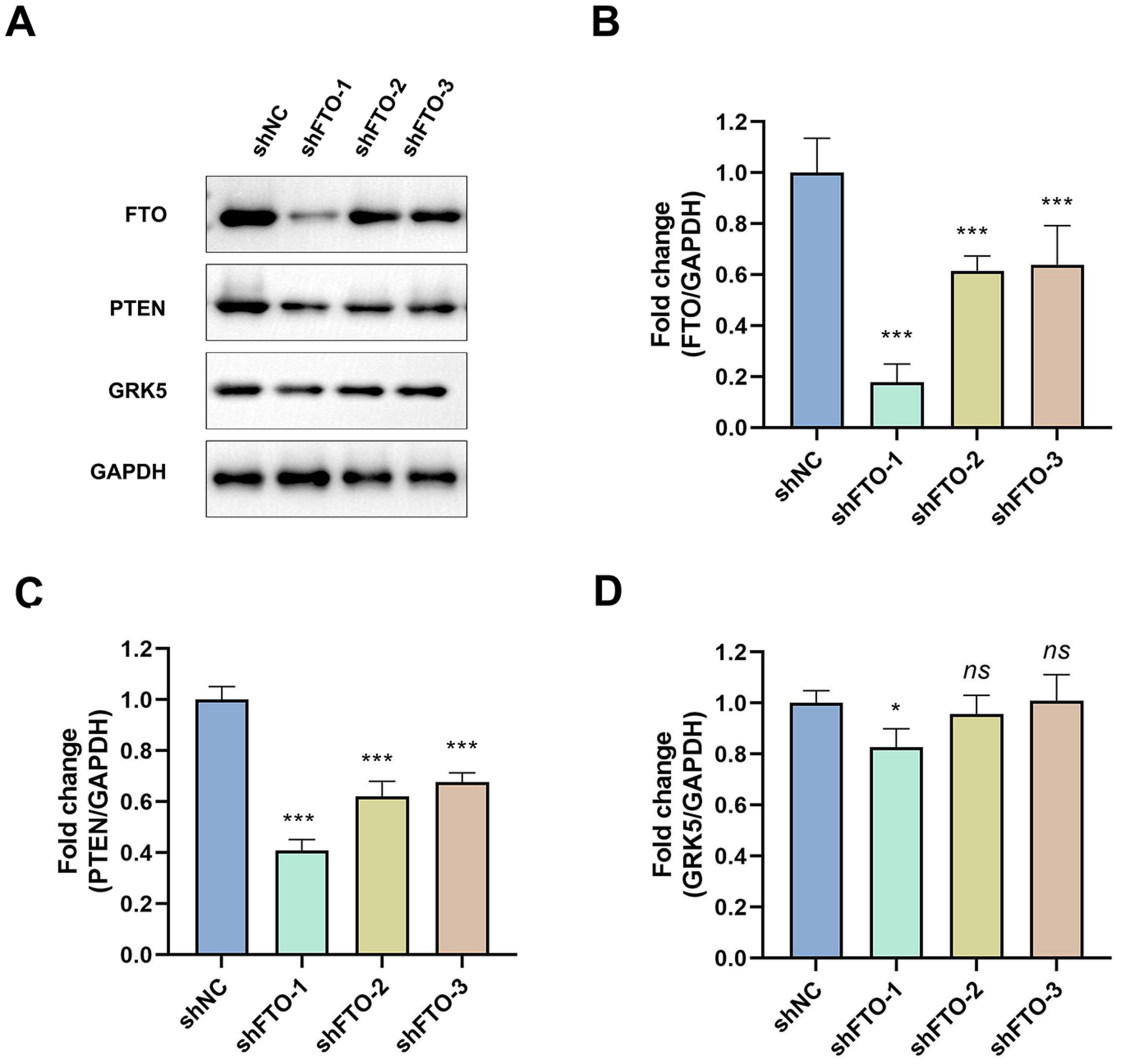
Western blot and quantitative analysis of PTEN and GRK5 protein expression after stable sh-FTO transfection in HASMCs. **(A)** Representative western blot bands of FTO, PTEN and GRK5 in sh-NC and sh-FTO groups; GAPDH was used as internal reference. **(B–D)** Quantitative grayscale statistics of FTO, PTEN and GRK5 protein relative expression levels, respectively. Statistical analysis: unpaired Student's t-test, data presented as mean ± SD, *N* = 3 independent biological replicates, *p* < 0.05.

To further verify the potential causal relationship between FTO downregulation and suppressed expression of PTEN and GRK5 in an *in vitro* functional model, we performed specific FTO knockdown in human aortic smooth muscle cells (HASMCs) using short hairpin RNA (shRNA). Western blot analysis demonstrated that PTEN protein abundance was markedly reduced in FTO-knockdown cells, consistent with the expression trend observed in clinical aortic tissues. Meanwhile, GRK5 protein expression also decreased, though the magnitude of reduction was milder compared with PTEN (Figure 8A). Quantitative grayscale analysis further confirmed these protein expression alterations (Figures 8B–D). Collectively, these cellular findings confirm that alterations in FTO expression directly modulate the protein abundance of the two downstream target genes. However, the specific signaling pathways mediating this regulator*y* axis remain unclarified and require further experimental validation.

## Discussion

Aortic coarctation is a life-threatening congenital heart disorder driven by aberrant SMC migration and progressive aortic fibrosis (2). Despite surgical advances, long-term complications persist, highlighting the need to define molecular and epigenetic mechanisms of CoA pathogenesis. Here, we report the first comprehensive m⁶A epitranscriptomic landscape of infantile CoA and uncover a novel FTO/m⁶A/PTEN-GRK5 regulator*y* axis driving disease progression.

A defining finding is global m⁶A hypermethylation in CoA tissues, accompanied by widespread hypermethylation events enriched in focal adhesion and ECM remodeling pathways. Focal adhesion signaling regulates SMC adhesion, migration, and ECM deposition—processes central to aortic wall fibrosis and luminal narrowing (9). Our results suggest that dysregulated m⁶A modification disrupts these pathways, promoting aberrant SMC behavior and CoA development. Integrated multi-omics further identified HMOX1, a gene linked to aortic aneurysm and hypertension (10), as a downregulated m⁶A target, implying its potential role in CoA-associated vascular complications.

Mechanistically, we demonstrate that FTO downregulation is the primary cause of m⁶A hypermethylation in CoA. FTO is a canonical m^6^A demethylase, and its downregulation increases m⁶A abundance in multiple diseases (11, 12). Consistent with these findings, our data show FTO expression is reduced in CoA tissues, directly driving m⁶A hypermethylation. The m⁶A reader YTHDF1 was also downregulated, which may modulate translation of methylated transcripts, though its functional role requires further investigation.

Our shRNA knockdown data confirmed that FTO depletion simultaneously represses PTEN and GRK5 in HASMCs. Published epitranscriptomic studies have characterized the FTO-PTEN regulator*y* axis in different cell types. Hu et al. found that FTO removes m^6^A modification to stabilize Mxd1, which binds the PTEN promoter and suppresses its transcription in microglia (13). Lin et al. described another regulatory mode, where FTO upregulates NEDD4 via m^6^A modification to promote PTEN ubiquitination and degradation in pancreatic tumor cells (14). The two studies demonstrate that FTO can modulate PTEN expression through distinct m^6^A-dependent pathways, consistent with the expression changes of PTEN observed in our aortic tissue and cell experiments.

As for GRK, relevant genetic research in East Asian diabetic populations showed GRK5 participates in insulin and vascular signal transduction (15). Reduced GRK5 expression releases the inhibitory effect on profibrotic GPCR signaling, which matches the fibrotic phenotype induced by FTO knockdown in our HASMC model. Existing m⁶A-related research mainly focuses on the interaction between FTO and PTEN, while few studies have discussed the regulatory relationship between FTO and GRK5.

The western blot results showed PTEN protein decreased more significantly than GRK5 after FTO silencing. This may be attributed to the different functional positions of the two genes in signaling cascades: PTEN is the core inhibitory molecule of the fibrotic PI3 K/AKT pathway and acts as the main downstream effector of FTO signaling, while GRK5 only serves as an auxiliary regulator of GPCR signaling. At present, we have not identified the specific m⁶A modification sites on GRK5 transcripts and the corresponding binding reader protein, and further MeRIP and dual-luciferase reporter experiments are needed to clarify this regulatory process.

### Limitations

This study has several limitations. First, the sample size for high-throughput sequencing is modest, requiring independent validation in larger cohorts. Second, functional roles of identified m⁶A targets (e.g., HMOX1) and regulators (e.g., YTHDF1) remain uncharacterized. Third, we only validated mRNA expression; protein analysis and functional assays are needed to confirm the regulator*y* axis. Future work will address these limitations.

## Conclusion

In summary, this work further supplements the downstream regulatory targets of FTO in vascular smooth muscle cells, and modulating FTO activity to recover PTEN and GRK levels may provide a potential direction for alleviating aortic fibrosis in infant CoA.

## Data Availability

The original contributions presented in the study are included in the article/Supplementary Material, further inquiries can be directed to the corresponding author.
