# Supplementary material for: N6-methyladenosine epitranscriptomic remodeling drives infantile aortic coarctation via FTO-mediated repression of PTEN and GRK5
Source: Front Cardiovasc Med. 2026 Aug 12;13:1882212. doi: 10.3389/fcvm.2026.1882212 (PMC13507676; doi:10.3389/fcvm.2026.1882212)
Supplement: Supplementary file 1 [file Datasheet1.docx]

**Surpporting imformation for**

**Comprehensive characterization of m^6^A modifications in congenital aortic constriction**

**Table S1: Primers used for Real-time Quantitative PCR**

| **Transcript** | **Forward (5’ to 3’)** | **Reverse (5’ to 3’)** |
| --- | --- | --- |
| PTEN | TGGATTCGACTTAGACTTGACCT | GGTGGGTTATGGTCTTCAAAAGG |
| SMAD3 | TGGACGCAGGTTCTCCAAAC | CCGGCTCGCAGTAGGTAAC |
| GRK5 | CCAACACGGTCTTGCTGAAAG | TCTCTGTCTATGGTCCTTCGG |
| SLIT3 | GTCAGCGTCATCGAGAGAGG | TTCGGCGTGCTCTGGAAAAG |
| SQSTM1 | GACTACGACTTGTGTAGCGTC | AGTGTCCGTGTTTCACCTTCC |
| METTL3 | CATTGCCCACTGATGCTGTG | AGGCTTTCTACCCCATCTTGA |
| METTL14 | GAACACAGAGCTTAAATCCCCA | TGTCAGCTAAACCTACATCCCTG |
| FTO | AACACCAGGCTCTTTACGGTC | TGTCCGTTGTAGGATGAACCC |
| ALKBH5 | ATCCTCAGGAAGACAAGATTAG | TTCTCTTCCTTGTCCATCTC |
| YTHDF1 | ACCTGTCCAGCTATTACCCG | TGGTGAGGTATGGAATCGGAG |
| YTHDF2 | CCTTAGGTGGAGCCATGATTG | TCTGTGCTACCCAACTTCAGT |

**Table S2 Top 27 differential expression IncRNAs in CAC**

| **Gene Name** | **Regulation** | **log2 (fold change)** | **p value** |
| --- | --- | --- | --- |
| AC016205.1 | up | 3.757121414 | 0.001936 |
| LUCAT1 | up | 2.395181050 | 0.016785 |
| AC093627.7 | up | 2.308226898 | 0.004193 |
| AL121656.1 | up | 1.995810621 | 0.010849 |
| AC142472.1 | up | 1.860887128 | 0.020575 |
| LINC02251 | up | 1.842233524 | 0.046561 |
| AC092910.3 | up | 1.805127077 | 0.003612 |
| LINC00847 | up | 1.399265345 | 0.011508 |
| MIRLET7BHG | up | 1.346033059 | 0.042293 |
| ILF3-AS1 | up | 1.304857821 | 0.006175 |
| LINC01896 | up | 1.205390766 | 0.003468 |
| AC013451.1 | down | -1.273915233 | 0.014398 |
| AC005332.9 | down | -1.374324789 | 0.001609 |
| SRP14-AS1 | down | -1.506931560 | 0.010767 |
| AL353708.3 | down | -1.600590735 | 0.000911 |
| AL669831.5 | down | -1.887685348 | 0.007076 |
| AC131025.2 | down | -1.931322461 | 0.010815 |
| AC110597.1 | down | -2.768732790 | 0.010751 |
| AC097381.1 | down | -2.785522303 | 0.025721 |
| LINC00158 | down | -2.900771626 | 0.039886 |
| AC237221.1 | down | -2.902026898 | 0.025886 |
| SAMMSON | down | -2.958337405 | 0.047873 |
| AC009961.1 | down | -4.000553022 | 0.028652 |
| AL354793.1 | down | -4.340704882 | 0.015163 |
| AC016588.2 | down | -4.377832309 | 0.008733 |
| SOX1-OT | down | -4.845347132 | 0.006267 |
| AP003086.1 | down | -5.017996079 | 0.001189 |

**Table S3 Top 40 differential expression mRNAs in CAC**

| **Gene Name** | **Regulation** | **log2 (fold change)** | **p value** |
| --- | --- | --- | --- |
| TEX14 | up | 8.206452927 | 0.000010 |
| GIMAP5 | up | 5.892697643 | 0.029820 |
| CPNE5 | up | 5.578958470 | 0.027893 |
| FGF7 | up | 5.023853233 | 0.001629 |
| PDIA2 | up | 4.614657436 | 0.009298 |
| CATSPERE | up | 4.538647408 | 0.010303 |
| SNX11 | up | 4.503983602 | 0.001040 |
| NPIPB2 | up | 4.337484835 | 0.002725 |
| MPV17L2 | up | 4.199368337 | 0.002238 |
| FYB2 | up | 4.019408415 | 0.035290 |
| ADRA1D | up | 4.015452949 | 0.004857 |
| VPS37C | up | 3.956859753 | 0.001870 |
| NKAIN1 | up | 3.956407946 | 0.007990 |
| KHK | up | 3.897641256 | 0.024912 |
| GRHL1 | up | 3.885825815 | 0.001804 |
| ABCA13 | up | 3.845895417 | 0.003774 |
| TIE1 | up | 3.766799265 | 0.029479 |
| PYROXD2 | up | 3.659153694 | 0.004409 |
| PNCK | up | 3.657742997 | 0.014784 |
| AKAP3 | up | 3.623814257 | 0.000005 |
| KIAA0895 | down | -3.188690893 | 0.003248 |
| GRK6 | down | -3.207390195 | 0.002710 |
| IQCH | down | -3.232913017 | 0.039363 |
| C11orf80 | down | -3.282707339 | 0.031698 |
| ISCA2 | down | -3.357421401 | 0.012997 |
| LRRC63 | down | -3.436421917 | 0.012783 |
| PAX3 | down | -3.437351291 | 0.023864 |
| KCNK17 | down | -3.500107644 | 0.046956 |
| PARL | down | -3.552233463 | 0.045654 |
| PREB | down | -3.613626413 | 0.000807 |
| TUBA4A | down | -3.674936074 | 0.017882 |
| RAB6C | down | -3.759043145 | 0.045040 |
| ABTB1 | down | -3.777843976 | 0.004154 |
| LYPLA2 | down | -3.862044819 | 0.009358 |
| HRK | down | -3.910151140 | 0.001181 |
| KCNK2 | down | -3.916919633 | 0.003045 |
| ARL6IP6 | down | -4.051342695 | 0.040759 |
| DEPDC4 | down | -4.208796400 | 0.039294 |
| KCNH4 | down | -4.217516264 | 0.023282 |
| KCNK13 | down | -4.230029588 | 0.012235 |

**Table S4 Top 20 differential m^6^A peaks of IncRNA in CAC**

| **Gene Name** | **Chromosome** | **Peak start** | **Peak end** | **Fold enrichment** |
| --- | --- | --- | --- | --- |
| LINC00982 | 1 | 3063417 | 3068437 | 828 |
| AC012676.1 | 16 | 4337188 | 4337698 | 709 |
| AP005329.2 | 18 | 3246775 | 3247277 | 557 |
| N4BP2L2-IT2 | 13 | 32506964 | 32507535 | 504 |
| AP005329.3 | 18 | 3246787 | 3247086 | 472 |
| LINC00472 | 6 | 71415536 | 71416046 | 460 |
| AL645608.1 | 1 | 920477 | 920807 | 437 |
| LINC01278 | X | 63427230 | 63428039 | 401 |
| AC022034.2 | 8 | 53516423 | 53523931 | 364 |
| MBNL1-AS1 | 3 | 152267493 | 152268691 | 338 |
| AL645608.1 | 1 | 920000 | 920359 | 321 |
| AL162377.1 | 13 | 51804402 | 51804938 | 321 |
| PAXIP1-AS2 | 7 | 154944133 | 154944639 | 320 |
| LINC00641 | 14 | 21204266 | 21204776 | 277 |
| LINC00667 | 18 | 5238394 | 5239054 | 277 |
| HYMAI | 6 | 144007544 | 144008262 | 256 |
| AC105020.4 | 15 | 75676226 | 75677162 | 254 |
| AL161421.1 | 13 | 48975382 | 48975799 | 252 |
| AP002761.3 | 11 | 73308676 | 73309361 | 231 |
| AC127496.6 | 17 | 80999650 | 81000130 | 224 |

**Table S5 Top 20 differential m^6^A peaks of mRNA in CAC**

| **Gene Name** | **Chromosome** | **Peak start** | **Peak end** | **Fold enrichment** | **Peak region** |
| --- | --- | --- | --- | --- | --- |
| MN1 | 22 | 27799043 | 27801498 | 3240 | CDS |
| OMD | 9 | 92415169 | 92424461 | 1910 | CDS |
| AOC3 | 17 | 42851273 | 42854143 | 1770 | CDS |
| SGMS2 | 4 | 107895556 | 107895976 | 1610 | CDS |
| DCHS1 | 11 | 6622341 | 6623152 | 1250 | CDS |
| RNF217 | 6 | 124962574 | 124963235 | 1240 | CDS |
| FAT4 | 4 | 125408701 | 125415781 | 1230 | CDS |
| ZBTB21 | 21 | 41990786 | 41991296 | 1190 | CDS |
| FOXC2 | 16 | 86567310 | 86569280 | 1180 | CDS |
| FLRT2 | 14 | 85621955 | 85624115 | 1160 | CDS |
| AKAP2 | 9 | 110136978 | 110137996 | 1150 | CDS |
| LPP | 3 | 188609188 | 188708450 | 1150 | CDS |
| PALM2-AKAP2 | 9 | 110136973 | 110137992 | 1060 | CDS |
| FAM208A | 3 | 56633181 | 56633572 | 970 | CDS |
| ZNF316 | 7 | 6653915 | 6654515 | 962 | CDS |
| MRAP2 | 6 | 84055362 | 84089685 | 874 | CDS |
| C1QTNF7 | 4 | 15442225 | 15443181 | 866 | CDS |
| ROR1 | 1 | 64159006 | 64179700 | 855 | CDS |
| BCL9L | 11 | 118901691 | 118902112 | 850 | CDS |
| F2R | 5 | 76716132 | 76716639 | 828 | CDS |

CDS: coding sequence
